# A qualitative account of young people’s experiences of alcohol screening and brief interventions in schools: SIPS Jr-HIGH trial findings

**DOI:** 10.1093/pubmed/fdz074

**Published:** 2019-08-21

**Authors:** E L Giles, G J McGeechan, S J Scott, R McGovern, S Boniface, A Ramsay, N Hendrie, E McColl, H Sumnall, D Newbury-Birch, E Kaner, the SIPS Jr-HIGH Study Team

**Affiliations:** 1 School of Health and Social Care, Teesside University, Middlesbrough, TS1 3BX, UK; 2 School of Social Sciences, Humanities and Law, Teesside University, Middlesbrough, TS1 3BX, UK; 3 Institute of Health and Society, Newcastle University, Newcastle, NE2 4AX, UK; 4 Institute of Psychiatry, Psychology, & Neuroscience, King’s College London, London SE5 8AF, UK; 5 Centre for Health Services Research, University of Kent, Canterbury, Kent, CT2 7NF, UK; 6 Faculty of Education, Health and Community, Liverpool John Moores University, Liverpool, L3 2 ET, UK

**Keywords:** alcohol, brief interventions, drinking, interviews, qualitative research, school, young people

## Abstract

**Background:**

The United Kingdom (UK) has seen a decrease in the number of young people drinking alcohol. However, the UK prevalence of underage drinking still ranks amongst the highest in Western Europe. Whilst there is a wealth of evidence reporting on the effectiveness of both primary, and secondary interventions, there are few reports of the experiences of young people who receive them.

**Methods:**

The present study reports findings from interviews with 33 young people who were involved in an alcohol screening and brief intervention randomized controlled trial in schools in England. All interviews were analysed using inductive applied thematic analysis.

**Results:**

Three major themes were identified following the analysis process: 1) drinking identities and awareness of risk; 2) access to support and advice in relation to alcohol use; and 3) appraisal of the intervention and potential impact on alcohol use.

**Conclusions:**

There appeared to be a reluctance from participants to describe themselves as someone who drinks alcohol. Furthermore, those who did drink alcohol often did so with parental permission. There was variation amongst participants as to how comfortable they felt talking about alcohol issues with school staff. Overall participants felt the intervention was useful, but would be better suited to ‘heavier’ drinkers.

## Introduction

Young people (aged between 10–24)^1^ are at particular risk from negative physical and psychological health effects due to alcohol consumption, which led to the Chief Medical Officer for England advising that those under the age of 15 should not drink alcohol at all.^2^ Evidence suggests that drinking alcohol before the age of 18 can impact on brain development, increase the risks of accidents and injury, teenage pregnancy, sexually transmitted infections,^3–5^ and is a strong prognostic indicator for the development of alcohol related issues in adulthood.^6^

The number of young people who have ever tried alcohol in the United Kingdom (UK) is declining. While 64% of young people aged 11–15 reported ever having tried alcohol in 2003, reducing to 44% in 2016.^7^ However, as young people become older, the level to which they consume alcohol increases,^7^ and those who do use alcohol are drinking more than people of the same age in the past.^8^ Furthermore, the prevalence of drinking among young people in England remains amongst the highest in Europe.^9^ Therefore, there are still those who remain at risk from their alcohol use, demonstrating a need for effective interventions.

Previous research suggests that the school is an ideal setting for delivering alcohol interventions,^10,11^ by providing access to a captive audience who^12^ are used to receiving health and social care education.^12^ Typically, in school settings, interventions tend to be primary prevention, aimed at the whole school population (e.g. in assemblies) regardless of individual consumption levels. Alcohol screening and brief interventions (ASBIs) are defined as a form of secondary prevention intervention, targeting individuals identified to be drinking alcohol at risky levels, which require limited curriculum time to deliver.^13^ Risky drinking is typically defined in relation to harmful drinking—levels of consumption that increase the chance of health issues developing; hazardous drinking—consumption which has already led to such issues and heavy episodic drinking—consuming more than six unit of alcohol in one sitting.^14^ While there is some evidence exploring the use of ABSIs for risky drinking among young people in other settings including emergency departments and universities, there is limited research in school settings.^15^ Furthermore, within these studies, there is a lack of exploration of the views of young people who have received targeted secondary prevention approaches; such views are required to understand the key mechanisms and acceptable processes that can lead to the effective implementation of these interventions.

This study formed part of a process evaluation of a large, multi-centre randomized controlled trial looking at the effectiveness and cost-effectiveness of a high school based alcohol screening and brief intervention.^16,17^

The aim of the present paper is to outline the findings from interviews with young people exploring their views of being involved in the trial and their views on any derived benefits, adverse events or improvements related to an ASBI delivered in a school setting.

## Methods

### Intervention

The authors have detailed the intervention elsewhere.^16,17^ To summarize, learning mentors working within schools were trained to deliver a screening questionnaire followed by either an alcohol brief intervention based on principles of motivational interviewing^18^ or treatment as usual. The screening questionnaire included lifestyle questions on smoking, energy drink consumption and risky sexual behaviour; questions related to well-being and the questions on alcohol consumption including the adolescent single alcohol question (A-SAQ) which is a modified version of the single alcohol-screening questionnaire.^19,20^

### Participant recruitment

To be eligible for the interviews, young people had to have agreed to be contacted about taking part in an individual interview and left their name on the screening questionnaire. Purposive sampling was employed to ensure representative recruitment in terms of sex, ethnicity, score on the A-SAQ (positive or negative), and study site (See Table 1 below). For the interviews, the aim was to recruit 10 young people from each of the four regions, five boys and five girls (40 in total). As the researchers were blinded to the study condition, it was not possible to recruit in line with trial arm. Therefore, for those participants who scored positive on the A-SAQ, we did not know prior to the interview whether or not they had received the ASBI.

**Table 1:** Young People Interview Sampling Framework.

Region		North East					North West				London				Kent				Total
A-SAQ		positive			negative		positive		negative		positive		negative		positive		negative		
Target		8			2		4		6		2		8		6		4		40
Recruited		7			1		3		4		1		7		6		4		33
Ethnicity																			
White	Target	6			0		3		1		1		1		4		2		17
		2 M	4 F		0 M	0 F	2 M	1 F	1 M	0 F	1 M	0 F	0 M	1 F	3 M	1 F	1 M	1 F	
	Recruited	5			0		2		0		0		1		4		2		14
		2 M	3 F		0 M	0 F	2 M	0 F	0 M	0 F	0 M	0 F	0 M	1 F	3 M	1 F	1 M	1 F	
Asian	Target	0			1		0		1		0		1		2		1		6
		0 M	0 F		1 M	0 F	0 M	0 F	0 M	1 F	0 M	0 F	1 M	0 F	0 M	2 F	0 M	1 F	
	Recruited	0			1		0		1		0		0		2		1		5
		0 M	0 F		1 M	0 F	0 M	0 F	0 M	1 F	0 M	0 F	0 M	0 F	0 M	2 F	0 M	1 F	
Black	Target	0			1		1		2		0		6		0		1		11
		0 M	0 F		0 M	1 F	0 M	1 F	1 M	1 F	0 M	0 F	3 M	3 F	0 M	0 F	0 M	1 F	
	Recruited	0			0		1		2		0		6		0		1		10
		0 M	0 F		0 M	0 F	0 M	1 F	1 M	1 F	0 M	0 F	1 M	5 F	0 M	0 F	0 M	1 F	
Mixed	Target	1			0		0		1		1		0		0		0		3
		1 M	0 F		0 M	0 F	0 M	0 F	0 M	1 F	0 M	1 F	0 M	0 F	0 M	0 F	0 M	0 F	
	Recruited	1			0		0		1		1		0		0		0		3
		1 M	0 F		0 M	0 F	0 M	0 F	0 M	1 F	0 M	1 F	0 M	0 F	0 M	0 F	0 M	0 F	
Chinese	Target	0			0		0		0		0		0		0		0		0
		0 M	0 F		0 M	0 F	0 M	0 F	0 M	0 F	0 M	0 F	0 M	0 F	0 M	0 F	0 M	0 F	
	Recruited	0			0		0		0		0		0		0		0		0
		0 M	0 F		0 M	0 F	0 M	0 F	0 M	0 F	0 M	0 F	0 M	0 F	0 M	0 F	0 M	0 F	
Other	Target	1			0		0		1		0		0		0		0		2
		1 M	0 F		0 M	0 F	0 M	0 F	1 M	0 F	0 M	0 F	0 M	0 F	0 M	0 F	0 M	0 F	
	Recruited	1			0		0		0		0		0		0		0		1
		1 M	0 F		0 M	0 F	0 M	0 F	0 M	0 F	0 M	0 F	0 M	0 F	0 M	0 F	0 M	0 F	

Schools participating in the trial acted as gatekeepers for the qualitative interviews. They distributed the information sheets and invitation letters to those young people who consented to be contacted about the interview (*n* = 206), young people then informed their learning mentors if they did not want to participate in the interviews. Learning mentors liaised with the research team to organize a suitable date and time for interviews to take place. In order to minimize impact on schools the 206 eligible young people were randomly allocated to the sampling framework, with each young person approached in turn. All of those young people who were approached for an interview (*n* = 33) agreed to take part and were subsequently interviewed. Ethical approval was obtained from the School of Health and Social Care Ethics and Research Governance Committee (047/16).

### Data collection

A standardized semi-structured interview guide was developed which asked a number of questions on the experiences of young people’s involvement in the ASBI and wider views on alcohol use.

All interviews were held on school premises. Researchers undertook the interviews with young people in their region. Written assent or consent was obtained immediately prior to the interview, dependent on whether the young person was aged 15 or 16 years old, respectively.

### Analysis

All interviews were recorded using a Dictaphone, and then transcribed verbatim. Any identifying information was removed from the transcripts to ensure anonymity. Interviews were subjected to applied thematic analysis^21^ with an inductive approach undertaken as we were not coding in line with theory. This allowed for a greater depth of experience to emerge as the analysis was not constrained by the researchers *a priori* assumptions.^22^

One researcher (GM) conducted initial coding of the transcripts, whilst a senior researcher (EG) second coded a proportion of the transcripts to ensure reliability of interpretation of the emergent themes.^23^ Any disagreement between coders was resolved through discussion. Final themes were agreed in consultation with the study’s qualitative steering group.

## Results

Whilst the initial aim was to recruit 40 participants, due to time constraints it was only possible to recruit 33 young people to these interviews. Of those who took part in the interviews, 20 were girls and 13 were boys. Interviews lasted on average 20 minutes (range = 8–36 minutes). Participant characteristics are outlined in Table 2.

**Table 2:** Participant characteristics—young people interviews.

Code	Gender	Age	Ethnicity	A-SAQ	Condition	Site
Young Person 1	Male	15	White	Positive	Intervention	Kent
Young Person 2	Female	16	White	Positive	Intervention	North East
Young Person 3	Male	16	White	Positive	Control	Kent
Young Person 4	Female	16	White	Negative	N/A	London
Young Person 5	Female	15	White	Positive	Control	North East
Young Person 6	Female	16	Asian	Negative	N/A	Kent
Young Person 7	Female	16	White	Negative	N/A	Kent
Young Person 8	Female	16	White	Positive	Control	Kent
Young Person 9	Female	15	Black	Negative	N/A	London
Young Person 10	Female	16	Black	Negative	N/A	Kent
Young Person 11	Male	16	White	Positive	Intervention	North East
Young Person 12	Female	16	Mixed	Negative	N/A	North West
Young Person 13	Female	15	Asian	Positive	Intervention	Kent
Young Person 14	Male	16	White	Negative	N/A	Kent
Young Person 15	Female	15	White	Positive	Control	North East
Young Person 16	Male	15	Mixed	Positive	Control	North East
Young Person 17	Female	Unknown	Asian	Negative	N/A	North West
Young Person 18	Male	16	White	Positive	Intervention	Kent
Young Person 19	Male	Unknown	Black	Negative	N/A	North West
Young Person 20	Female	15	Black	Negative	N/A	London
Young Person 21	Male	16	White	Positive	Control	North East
Young Person 22	Male	Unknown	White	Positive	Intervention	North West
Young Person 23	Female	16	Black	Negative	N/A	London
Young Person 24	Female	16	Asian	Positive	Control	Kent
Young Person 25	Male	15	Other	Positive	Intervention	North East
Young Person 26	Male	16	White	Positive	Control	North West
Young Person 27	Female	16	Black	Negative	N/A	London
Young Person 28	Male	15	Asian	Negative	N/A	North East
Young Person 29	Female	Unknown	Black	Negative	N/A	North West
Young Person 30	Male	15	Black	Negative	N/A	London
Young Person 31	Female	16	Black	Negative	N/A	London
Young Person 32	Female	15	Black	Positive	Control	North West
Young Person 33	Female	16	Mixed	Positive	Control	London

Following analysis, three themes were identified: 1) drinking identities and awareness of risk; 2) access to support and advice in relation to alcohol use; and 3) appraisal of the intervention and potential impact on alcohol use. Illustrative quotations are provided below within each theme.

### Drinking identities and awareness of risk

Most of the young people clearly identified themselves within the interviews as non-drinkers. Although some said that they did drink alcohol, they did not self-identify as drinkers, instead identifying ‘others’ who drank more than they did as such. This suggests that young people draw a sense of identity from their level of consumption and there is perhaps a level of stigma associated with being a ‘drinker’.

*‘I think if I was drinking a lot more, or a little bit more, it would definitely change it, but because I don’t drink that much, it [intervention] didn’t have too much of an effect.’*—Young Person (YP) 1

Participants were able to identify potential immediate risks associated with drinking at younger ages, particularly accidents and being separated from friends. However, some did also consider longer-term risks to health associated with initiating alcohol consumption at a young age.

‘You know that you’re on your way to being drunk? That’s when I know that I should stop. I wouldn’t feel safe. If I had to walk home by myself, I wouldn’t feel safe.’ YP15

*‘I think it was something to do with like, I can’t remember what it was, but it can lead to cancer and stuff and there’s just loads of illnesses that you can get from it.’*—*YP3*

The social side of drinking was highlighted by many young people, alongside the extra confidence that drinking alcohol provided. Drinking was seen as a fun activity, and a way to de-stress, particularly at exam time and as part of a shared social identity. Those who did not conform could be subjected to peer pressure, a common reason offered by participants as to why they consumed alcohol. Such peer pressure seemed to come from wider social circles rather than from their immediate friendship group.

*‘So it may be, if you don’t do it, you feel, like you’re going to be judged by your friends, judged by others, or, another, reason why you may go drink out, like you go out to parties and, having the like, forced to drink, is, because maybe people, who are this age, think it’s cool to drink.’*—*YP4*

*‘I can just go out and like only two people drink, if they want to drink they can, some people don’t want to, so we’re not bothered.’*—*YP5*

Inter-generational influences were said to have an impact on whether the participants consumed alcohol. Some participants only drank alcohol with parental agreement, usually for special occasions. That said, this parental influence could vary depending on cultural differences, with one participant in particular, from an Asian background, describing strict rules within their household relating to alcohol consumption. Furthermore, even with parental consent, there was a recognition that consumption needed to be within certain boundaries, and if they drank too much alcohol there would be repercussions. This trust between parents and young people allowed them to experiment with alcohol, but within a safe context.

*‘She [mother] doesn’t like it. Sometimes like if I was at a party she’d go like in the shop for me and get me something to drink so she’d know what I was drinking and stuff but then if I was just saying I was drinking in the street or I drank like two days in a row then she’d say (name), don’t drink anymore.’*—*YP2*

*‘My parents do not allow drinking, but English people, I shouldn’t say this, English people might find it alright to let the children drink at certain age, like 16 is alright.’*—*YP6*

### Access to support and advice on alcohol use

When discussing support and advice on alcohol use a distinction could be drawn in terms of formal support—that provided by individuals or organizations trained to offer such advice; and informal support—that delivered by peers or family members. In terms of formal support, young people felt there was a lack of guidance within the school. They acknowledged that schools provided general assemblies on alcohol, as well as one-off sessions within Personal, Social, Health and Economic Education (PSHE) lessons. However, they perceived it to be out-dated, and that it did not apply to them.

*‘No, it does pop up in assembly, like, a couple of times, but they don’t go into depth about it. Just like kind of mention it and then go onto something else.’*—*YP7*

*‘I believe that erm young people should be aware of the drinking and but I don’t feel like what they are giving us is effective enough. I feel like it is just a waste of time, a waste of their money, a waste of our valuable education.’*—YP9

As support and advice provided by schools were viewed as insufficient, participants pursued other sources of support. For example, young people sought advice from older friends, and family members. However, there were mixed feelings when it came to speaking to parents, very much depending on whether or not they allowed alcohol consumption. Those who did not discuss alcohol with their parents were perhaps fearful of repercussions from doing so, instead seeking advice from another family member or friend.

*‘And I’ve also had some advice from my brother as well, he says to make sure you go out with people you’re actually good friends with so they don’t leave you if you’re really drunk.’*—YP10

*‘Probably like my stepdad, my stepdad’s kind of got the whole mentality of like, he kind of realized what it was like because he did it when he was my age and he could probably help me with it.’*—YP11

### Appraisal of the intervention and potential impact on drinking behaviour

Those participants randomized to the intervention arm of the trial thought it would have limited impact on their own drinking. The effectiveness of the intervention was linked to their identity—or not—as a drinker and feeling that because they did not drink a lot of alcohol they did not require support. That said, some of the young people who received the intervention did state that they had consequently made some small changes.

*‘I was really confused. I was like, ‘Why am I doing this?’ The closest I’ve got to something relating to alcohol is probably sparkling water.’*—YP12

*‘So then like from the session, so I wanted to do it ‘cause like it made me understand why I shouldn’t do it and supported me in thinking why I should probably drink less. She was like you shouldn’t drink that much because these are the reasons why, so basically it supported me into like reducing how much I drink, yeah.’*—YP13

Most young people who received the intervention said that they were comfortable discussing alcohol with learning mentors, and felt reassured that the sessions were confidential. However, some students were aware that no matter how informal their relationship with learning mentors was, mentors were still school staff. This led to a fear amongst some that fully disclosing the level of their alcohol use could lead to negative consequences, and as such, some students withheld information.

*‘Because I think, I knew that no one would be told about it and it’s like personal and secretive so I knew like my name wouldn’t be like going around and like and that it wouldn’t be broadcast.’*—YP13

*‘I just don’t think it’s one of those things that you’d really want to go and speak to someone about like going and talking to* (name), because (name’s) *nice but I wouldn’t feel comfortable just sitting there and telling him everything about me, do you know what I mean?’*—YP16

## Discussion

### Main findings of the study

Within this study, it was clear that young people were wary of identifying themselves as ‘drinkers’. However, it must be noted that those randomized to the intervention, who described themselves as non-drinkers, had scored positively on a validated measure for risky drinking.^24^ This incongruity could be linked to social desirability bias, or that they were unwilling to fully disclose alcohol use to school staff. There was perhaps some stigma attached to being identified as an underage drinker.

While many of the young people could identify both short- and long-term risks associated with consuming alcohol, they tended to focus more on the short-term risks. Additionally, parental influences were identified as important in determining whether young people drank alcohol. Often, young people were only permitted to drink with parental consent, as part of a trusting relationship.

Young people found it acceptable to receive an alcohol brief intervention in school; however, there was concern around discussing their alcohol use with school staff. This may be indicative of an unwillingness to self-identify as a drinker.

### What is already known on this topic?

Research indicates that young people (11–15) associate more with a prototype of a ‘non-drinker’^25^ however the term non-drinker can mean different things to different young people and may be related to the dominant norms within their social group such that ‘non-drinkers’ can be on a continuum ranging from tee-total to occasional drinker.^26^ Furthermore, research has highlighted that in order for young people to fit in with their peers then they must keep up with social norms,^27^ suggesting that they may model their consumption levels based on their peer group yet apply labels to their behaviour based on dominant norms.^28,29^ However, other studies have suggested that those who self-identify as occasional, or light drinkers are actually at high risk of harm.^30^ This suggests that young people’s reluctance to self-identify as a drinker may be more to do with social identity, and unrealistic optimism than actual levels of consumption.^7,31,32^

Parental influence has implications for reducing alcohol consumption by young people, in that the targets of health promotion interventions may not always need to be the young people themselves; rather, targeting parents may also be required.^33,34^ Parents and families are important influences on adolescents and factors such as communication about alcohol use, parental modelling of behaviour, parental monitoring/supervision of free time, and availability of alcohol have all been shown to effect children’s own alcohol use. Furthermore, as touched on in our results there may be cultural differences in alcohol use such that children from Asian or African backgrounds may face greater parental monitoring and stronger disapproval of consumption compared to White British children. This could then lead to a barrier in accessing services for some ethnic minority children who do drink, for fear of reprisal.^35^

Some parents may be willing to supply alcohol to their children in an attempt to minimize harm,^36,37^ feeling that they would probably drink alcohol anyway, and a desire not to isolate them from their peers.^38^ However, research suggests that parental supply of alcohol can be associated with increased drinking episodes and harms amongst young people.^39^

Within this present study, we aimed to recruit a number of parents to interviews to discuss their view on the intervention. However parents proved to be an extremely hard group to recruit, as found in previous work.^15^ Nonetheless, given that many of our participants drank with their parents’ permission and the potential adverse impact of parental supply, parents remain a potentially valuable component in addressing adolescent alcohol consumption. Indeed a recent review of combined school and parental interventions highlighted their potential efficacy as parents and families can play a key role in influencing and delaying alcohol use in young people. This can be achieved by setting clear rules on alcohol use, encouraging open and honest dialogue to provide informal support to young people and by limiting parental supply.^40^

Previous research looking at the effectiveness of alcohol screening and brief interventions has been limited to primary prevention models, with limited research into the effectiveness of secondary prevention.^10,11,41^ Therefore, it is important to explore young people’s experiences of such interventions to ascertain whether they are a feasible model of delivery.^15,42^ Previous evidence has also highlighted that a lack of trust between young people and those delivering an intervention can be a barrier to effectiveness.^43,44^ Further evidence suggests that young people may be more willing to engage with a digital intervention which would potentially resolve this barrier.^4,45^

### What this study adds

To the best of our knowledge, this is the first study to explore young people’s experiences of an individually randomized ASBI delivered in schools in England.^19^ The results of this study have highlighted that the students’ informal relationship with learning mentors may have facilitated discussions with staff about alcohol use. However, some students admitted to withholding information, as they did not feel comfortable discussing alcohol use with school staff.

Health promotion messages often tend to focus on adopting short- to medium-term healthy behaviours to result in longer-term health benefits.^46^ In this respect, it may be important with this age group that such health promotion messages and interventions focus more on the short- to medium-term risks, or the issues prevalent to young people such as being separated from friends on a night out, which may help deliver more appropriate harm minimizing messages.^32,47,48^ The results of this study indicate that young people are willing to receive advice and support in relation to alcohol use, however formal support offered in schools is outdated and does not resonate with them. Instead, they seek more informal advice from friends and family. As evidence suggests parental involvement in such interventions can be effective then future research should focus on how to better involve parents in school-based services and/or interventions.^40^

### Limitations of this study

Whilst we aimed to recruit a purposive sample of participants from across the four study sites, this was not possible due to regional variations in recruitment to the trial as a whole. Furthermore, whilst we aimed to recruit 40 participants, we were only able to interview 33 young people due to time constraints within schools. However, no regional specific issues were identified and data saturation was reached. Finally, the length of the interviews was quite short, averaging 20 minutes however, this is consistent with previous qualitative research with children.^49^
